# The role of the medial prefrontal cortex in cognition, ageing and dementia

**DOI:** 10.1093/braincomms/fcab125

**Published:** 2021-06-11

**Authors:** Dan D Jobson, Yoshiki Hase, Andrew N Clarkson, Rajesh N Kalaria

**Affiliations:** 1 Translational and Clinical Research Institute, Newcastle University, Campus for Ageing & Vitality, Newcastle upon Tyne NE4 5PL, UK; 2 Department of Anatomy, Brain Health Research Centre and Brain Research New Zealand, University of Otago, Dunedin 9054, New Zealand

**Keywords:** ageing, default mode network, dementia, prefrontal cortex, vascular cognitive impairment

## Abstract

Humans require a plethora of higher cognitive skills to perform executive functions, such as reasoning, planning, language and social interactions, which are regulated predominantly by the prefrontal cortex. The prefrontal cortex comprises the lateral, medial and orbitofrontal regions. In higher primates, the lateral prefrontal cortex is further separated into the respective dorsal and ventral subregions. However, all these regions have variably been implicated in several fronto-subcortical circuits. Dysfunction of these circuits has been highlighted in vascular and other neurocognitive disorders. Recent advances suggest the medial prefrontal cortex plays an important regulatory role in numerous cognitive functions, including attention, inhibitory control, habit formation and working, spatial or long-term memory. The medial prefrontal cortex appears highly interconnected with subcortical regions (thalamus, amygdala and hippocampus) and exerts top-down executive control over various cognitive domains and stimuli. Much of our knowledge comes from rodent models using precise lesions and electrophysiology readouts from specific medial prefrontal cortex locations. Although, anatomical disparities of the rodent medial prefrontal cortex compared to the primate homologue are apparent, current rodent models have effectively implicated the medial prefrontal cortex as a neural substrate of cognitive decline within ageing and dementia. Human brain connectivity-based neuroimaging has demonstrated that large-scale medial prefrontal cortex networks, such as the default mode network, are equally important for cognition. However, there is little consensus on how medial prefrontal cortex functional connectivity specifically changes during brain pathological states. In context with previous work in rodents and non-human primates, we attempt to convey a consensus on the current understanding of the role of predominantly the medial prefrontal cortex and its functional connectivity measured by resting-state functional MRI in ageing associated disorders, including prodromal dementia states, Alzheimer’s disease, post-ischaemic stroke, Parkinsonism and frontotemporal dementia. Previous cross-sectional studies suggest that medial prefrontal cortex functional connectivity abnormalities are consistently found in the default mode network across both ageing and neurocognitive disorders such as Alzheimer’s disease and vascular cognitive impairment. Distinct disease-specific patterns of medial prefrontal cortex functional connectivity alterations within specific large-scale networks appear to consistently feature in the default mode network, whilst detrimental connectivity alterations are associated with cognitive impairments independently from structural pathological aberrations, such as grey matter atrophy. These disease-specific patterns of medial prefrontal cortex functional connectivity also precede structural pathological changes and may be driven by ageing-related vascular mechanisms. The default mode network supports utility as a potential biomarker and therapeutic target for dementia-associated conditions. Yet, these associations still require validation in longitudinal studies using larger sample sizes.

## Teaser

Jobson et al. convey that the medial prefrontal cortex functional connectivity in man exhibits disease-specific alterations across dementia associated disorders. These abnormalities appear to precede structural changes and may be driven by ageing-related vascular mechanisms. This mainly affects the large-scale default mode network, thus providing potential biomarkers and therapeutic targets.

## Graphical Abstract

**Graphical Abstract:**
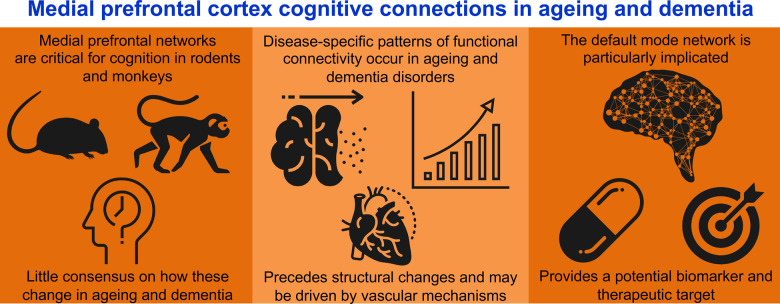

## Introduction

Greater understanding of the significance of the prefrontal cortex (PFC) is probably owed to the serendipitous discovery after the unusual accident suffered by Phineas Gage in 1848. The iron-tamping rod he had used on the railroad had pierced through his orbitofrontal lobe and changed him forever from once a respectable family man quickly into an ill-tempered irrational individual. We now know that a plethora of higher cognitive skills in order to perform crucial executive functions, such as reasoning, planning, language and social interactions, are regulated predominantly by the PFC which contains the orbitofrontal region.^1^ Through observing humans and other primates with specific PFC lesions, we now appreciate precise locations are associated with deficits. For example, the dorsolateral PFC (dlPFC) is associated with planning, strategy building and executive decisions, whereas the orbitofrontal region is related to inhibiting primal survival responses arising with the limbic system (glossary, Box 1). The PFC also appears to be involved in emotional states through extensive connections to areas controlling release of the mood-altering biogenic amines, including dopamine, noradrenaline and serotonin.^2^ Specific regions of the PFC have been implicated in a variety of neurocognitive disorders revealed by neuroimaging studies in life and by post-mortem brain research. The PFC typically refers to the granular (glossary, Box 1) and orbital aspects of the frontal cerebral cortex receiving reciprocal projections from the mediodorsal nucleus of the thalamus according to Rose and Woolsey’s anatomical studies in mammals.^3–5^ However, later studies showed that the mediodorsal nucleus of the thalamus does not project exclusively to the PFC and that other thalamic nuclei, such as the reuniens and rhomboid nuclei, display PFC projections. These advances indicate that there still appears a lack in satisfactorily identifying the PFC with clear homology across all species.^6^ However, the PFC is typically suggested as the region anatomically located anterior to the premotor cortex and supplementary motor area.^7^

**Box 1:** Glossary: key and unfamiliar terms used with their respective definitions

Term	Definition
Agranular	Brain regions lacking neocortical layer IV
Amyloid-β	Primary component of plaques found in Alzheimer's disease
*APOE4*	Protein that metabolizes fats as an Alzheimer's disease risk factor
Brain atrophy	Loss of neurons and connections between them
Brodmann areas	System to divide the cerebral cortex into regions
Cognitive function	Mental processes that allow us to carry out tasks
Continuous performance task	Test that measures sustained/selective attention in humans
Cytoarchitectonic	The microscopic study of cellular composition
Default mode network	Interacting brain regions that activate during rest
Diaschisis	Impaired brain function in one region due to localized damage in another connected area
Effective connectivity	Causal influence neural units exert over another
Endothelin-1	Secreted peptide that is a potent vasoconstrictor
Executive control network	Interacting brain areas key for executive function
Fronto-parietal network	Interacting brain areas that initiate new task states
Frontotemporal lobar degeneration	Syndrome with progressive behaviour or language decline due to frontal/temporal lobe deterioration
Functional connectivity	The temporal correlation of time series between different brain regions
Graph theory	A method used for the mathematical study of fMRI networks
Granular	Brain regions containing neocortical layers I-VI
Heteromodal region	A region that receives inputs from multiple areas
Hoehn and Yahr scores	Scale describing Parkinson's disease motor symptom progression
Independent component analysis	A data-driven method used to analyse fMRI data
Iowa Gambling Task	A task used to measure human decision-making abilities
Limbic system	Cortical structures involved in memory and mood
Magnetoencephalography	Neuroimaging technique that identifies brain activity by measuring small magnetic fields
Neocortex	Area involved in higher sensory/motor functions
Object location recognition task	Task that requires rodents spatially remembering objects
Optogenetic	Technique that controls exact neural circuits live
PET	Neuroimaging technique used for measuring metabolic processes in the body
Photothrombosis model	Stroke model in rodents causing ischaemic damage in certain cortical areas
Principal sulcus	Superficial feature of the macaque dlPFC surface
Reinforcer devaluation task	Decision-making task in animal models whereby the food reinforcer value is reduced after cue completion
rs-fMRI	Neuroimaging technique to measure blood flow changes that occur with resting brain activity
Salience network	Interacting brain areas that detect salient stimuli
Seed-based	Finds regions correlated with chosen area activity
Structural connectivity	White matter tracts physically connecting regions
Tau pathology	Tau protein aggregation as neurofibrillary tangles
Voxel-based lesion-symptom mapping	fMRI method to analyse the tissue damage and behaviour association voxel-by-voxel

Brodmann gave the first topographical description of the ‘frontal’ and ‘precentral’ regions of the primate frontal lobe, which possessed a definitive granular pyramidal layer IV as a prominent characteristic. Although the anterior cingulate cortex (ACC), which contains agranular (glossary, Box 1) aspects that lack layer IV is often included within the PFC, since this structure additionally receives mediodorsal nucleus of the thalamus inputs. Based upon cytoarchitectonic (glossary, Box 1) and topographical criteria widely used within primates, the Brodmann areas (BAs) (glossary, Box 1) that typically define the PFC in humans include BA8 to 14 and BA44 to 47.^6^ In addition, the PFC can be divided into two generalizable regions based upon neuroanatomical connections: the medial prefrontal cortex (mPFC) and lateral prefrontal cortex (lPFC), which can be further separated into respective dorsal and ventral subregions. Some investigators divide the PFC into two broad regions mainly related to their functions: the dlPFC and the ventromedial PFC, which is also referred to as the orbitofrontal PFC. The PFC is also thought to contain three separate, yet interconnecting circuits responsible for specific aspects of memory, executive function and social behaviour: the dlPFC, the ACC and the orbitofrontal cortex, each which is associated with different functions but share a similar cortico-subcortical framework, originating in the PFC before projecting to respective aspects of the caudate-putamen before reaching the globus pallidus and substantia nigra, then ultimately connecting to the thalamus before the circuit is completed by reverting back to the PFC. Much of what is known about the function of these circuits is through loss of function studies. Here, we focus on the mPFC that appears to have connections with the amygdala, hippocampus (ventral) and temporal areas, which integrates information from environmental stimuli^8^; whereas the lPFC has reciprocal projections with the basal ganglia, cingulate cortex and parietal cortex areas in order to regulate responses from environmental stimuli.^1^

The PFC has been theorized as involving top-down control by connecting other brain regions so as to enable complex cognitive processes, such as executive function.^9^ The umbrella term executive function has numerous definitions, but a common explanation may include the involvement of multifactorial higher-order cognitive processes that enable a person to perform independent, purposive and goal-directed behaviour. Thus, a wide range of cognitive operations is often reported as working together to constitute features, such as planning, verbal reasoning, problem-solving, resistance to interference, multitasking, cognitive flexibility, inhibitory control, decision-making, sequencing, working memory and the ability to maintain sustained attention and cope with novelty.^10–13^ Whilst substantial knowledge of individual PFC sub-division functions have been gained by assessing humans with brain damage, rodent and non-human animal models have also been crucial for investigating distinct structure–function relationships within the PFC through behavioural testing.^10^

**Figure 1:**
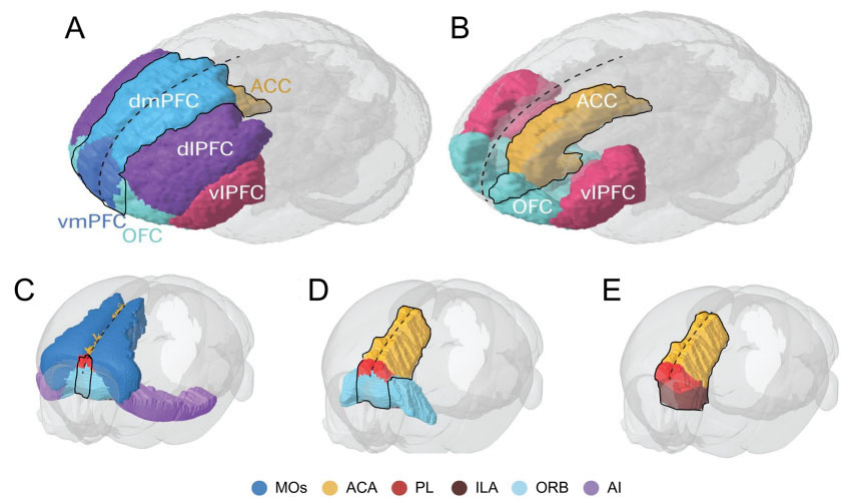
**Functional divisions of the human, non-human primate and rodent (mouse) prefrontal cortex** (**A and B**) Frontal-side view of the human primate brain with illustration of the prefrontal cortex functional divisions including the ACC, demarcated around the typically reported mPFC subregions of dmPFC, vmPFC and medial OFC. (**C–E**) Tilted frontal-side view of the rodent mouse brain illustrated with the agranular prefrontal cortex divisions and demarcated around the commonly stated mPFC subregions of ACA, PL, ILA and medial ORB. Dashed black line marks the sagittal midline. ACA, anterior cingulate area; ACC, anterior cingulate cortex; AI, agranular insular area; dlPFC, dorsolateral prefrontal cortex; dmPFC, dorsomedial prefrontal cortex; ILA, infralimbic area; MOs, secondary motor area; OFC, orbitofrontal cortex; ORB, orbital area; PL, prelimbic area; vlPFC, ventrolateral PFC; vmPFC, ventromedial prefrontal cortex. The schematic is adapted from Carlén.^6^

One such area that has been extensively implicated in the rodent literature regarding cognitive functions (glossary, Box 1) is the mPFC (Fig. 1). This region is often further divided into subregions that comprise the dorsomedial (dmPFC) and ventromedial PFC (vmPFC), primarily due to differences in cytoarchitecture and connectivity to other brain regions. The dmPFC, therefore, includes the medial pre-central cortex, the dorsal ACC and occasionally the dorsal aspects of the prelimbic PFC (plPFC) and ventral areas of the ACC. Whereas, the vmPFC can be subdivided into the more ventral parts of the plPFC in some instances, the infralimbic (ilPFC) as well as the medial orbital cortices (MO). Furthermore, the dmPFC has been attributed with major connections to the neocortex (glossary, Box 1), whilst the vmPFC has connections predominantly with the limbic system and both areas project to differing regions of the caudate/putamen within the sub-cortical basal ganglia structure. Therefore, it can be argued that some regional homologies are present between rodents and primates in terms of mPFC components, which reflect respective BAs.^14^^,^^15^

The PFC field itself is vast with a plethora of studies that have attempted to establish the distinct cognitive brain functions of these specific cortical subregions largely through neurochemical lesion and electrophysiological recording work in rodents as well as non-human primates, thereby complementing the elucidation of the human PFC in cognition. Yet, this has been somewhat contradicted by disparities in anatomy and functional homologies between species, as the supposed mouse PFC is composed anatomically different to primates with fewer, completely agranular areas in the frontal lobes (cf. Fig. 1). However, rodent models have enabled the study of the facets of executive function, neurons involved in executive circuit control and prefrontal pathology,^16^^,^^17^ since mPFC lesions leading to cognitive impairment have been associated with various human brain disorders, such as those arising from stroke or trauma as well as those of neurodegenerative origin.^18^ In addition, a range of mPFC network aberrations have been reported in humans on a larger scale in those with specific mPFC damage through neuroimaging paradigms, which may be a direct result of ageing and neuropathological processes.^19^ Therefore, focusing upon the mPFC from animal or human-based studies and how they differ between species may enable clearer understanding of the exact contributions of this lesser studied PFC sub-region or closest equivalent and the comparative changes in pathophysiological processes including the microvasculature. The dissection of its structural organization and neural circuit functions has fundamental implications for understanding the pathology and developing therapeutic strategies against neurological diseases affecting the PFC.

Here, we first discuss the experimental evidence from rodent and non-human primate studies, which attempt to decipher the role of mPFC within certain elements of cognition including working memory, decision-making, cognitive flexibility and attention. We next highlight key issues regarding the disparities of anatomy and function that currently exist between the rodent and primate work. Then, we convey experimental evidence of pathophysiological rodent models of ageing and dementia-associated neurological conditions, followed by an overview of mPFC connectivity in healthy subjects. We finally elucidate the functional connectivity (FC) (glossary, Box 1) differences in ageing and dementia-associated disorders in relation to vascular changes measured by resting-state functional magnetic resonance imaging (rs-fMRI) (glossary, Box 1). Our review reveals the crucial role that the mPFC portrays from a vascular perspective in a range of cognitive functions. This is pertinent to the vast range of mPFC connections to subcortical structures involved in several common dementias.

## Cognition and the mPFC in rodents and non-human primates

Utilizing an animal model for representing the complex aspects of human cognition has previously been postulated as being potentially ambiguous, which may be due to the imperfect homology of PFC subregions better reflecting more basic sensory and motor-related brain functions instead. Nevertheless, understanding the neurobiological basis of cognitive function in rodents and non-human primates is arguably still very useful by providing a simpler system, whilst retaining many complex characteristics of executive function domains (Table 1). Therefore, rodent and non-human primate models serve an essential role in acquiring functional evidence for divergent cognitive processes performed by anatomically distinct mPFC subregions.^10^^,^^15^

**Table 1:** Salient points discovered from rodent and non-human primate mPFC studies

Executive functions	Rodents	Non-human primates
Working memory	mPFC lesions show deficits for delayed response and (non)-matching-to-sample; EP shows a mixed picture, but spatial/outcome-related neuronal activity is important; ventral hippocampus has connectivity with mPFC	dlPFC lesion/damage shows deficits in delayed response and alteration tasks; EP shows delay-period activity from dlPFC or lPFC and spatial/non-spatial appears processed across the whole lPFC
Decision-making	OFC lesions show RDT impairment, mPFC lesions affects choice value processing during DD and OFC/mPFC are both necessary for uncertainty-based decision-making tasks; OFC update and compare choice values; amygdala/dorsomedial striatum has been shown to connect to the mPFC	MO lesions may affect RDT and the lPFC is implicated in primates; ACC encodes option values into future plans of action.
Cognitive flexibility	mPFC lesions impair EDS, whilst ACC lesions impair IDS during the attentional set-shifting task; set-shifting ability is also disrupted in mice after mPFC damage; mPFC lesion impairs reversal learning during complex image presentation via touchscreen task, whilst OFC damage impairs discriminative reversal learning abilities; dorsomedial thalamus/ventromedial striatum has been implicated to connect to the mPFC	A Wisconsin Card Sorting Test analogue shows that lPFC lesions produced EDS deficits; OFC lesions have additionally been shown to display premature deficits upon stimulus reversal
Attention	mPFC lesion impairs ability to perform 5-CSRTT, with the dmPFC likely mediating attentional function, whilst ilPFC monitors inhibitory actions instead with maximal performance requiring mPFC sub-regions’ distinct functions to interact together; EP evidence implies that plPFC and ACC regions may mediate preparatory attention and ilPFC controls impulsivity; subthalamic nucleus connects to the mPFC	Primates implicate involvement of mPFC as well as lPFC regions for differing aspects and types of attentional function including endogenous visual/auditory, preparatory and spatial; a Cambridge Neuropsychological Test Automated Battery touchscreen version of 5-CSRTT has been developed for use in non-human primates

5-CSRTT, 5-choice serial reaction time task; ACC, anterior cingulate cortex; DD, delay discounting; dlPFC, dorsolateral prefrontal cortex; dmPFC, dorsomedial prefrontal cortex; EP, electrophysiology; EDS, extra-dimensional shift; ilPFC, infra-limbic prefrontal cortex; IDS, intra-dimensional shift; lPFC, lateral prefrontal cortex; MO, medial orbital prefrontal cortex, mPFC, medial prefrontal cortex; OFC, orbitofrontal cortex; plPFC, pre-limbic prefrontal cortex RDT, reinforcer devaluation task; vlPFC, ventrolateral prefrontal cortex.

**Figure 2:**
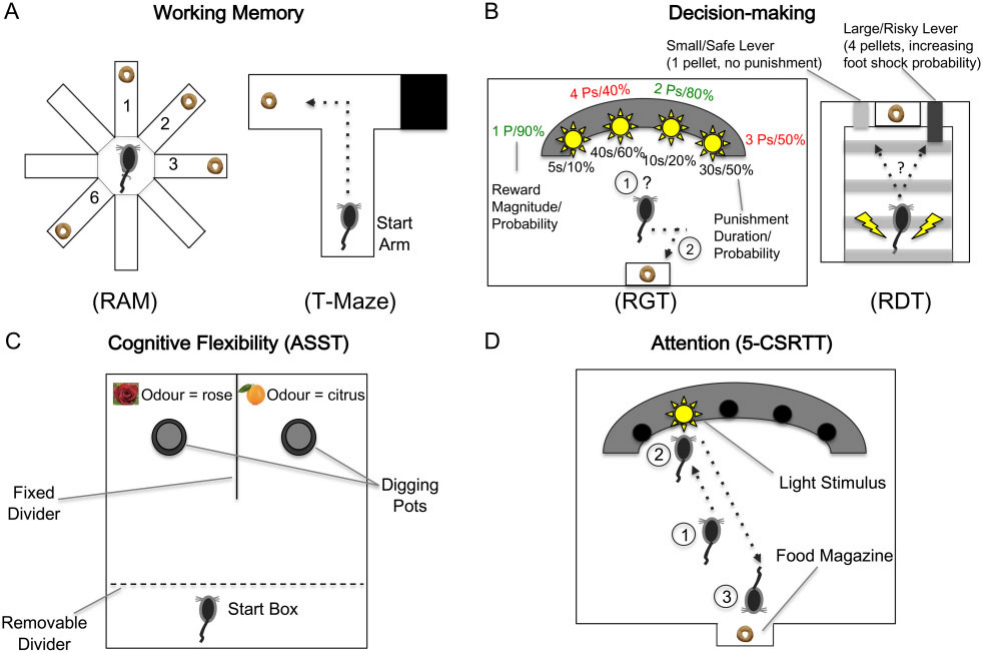
**Rodent behavioural paradigms tasking distinct cognitive domains of working memory, decision-making, cognitive flexibility, and attention** (**A**) The radial arm maze (RAM) and T-maze tasks assess working memory with delays and changing reward locations between trials. (**B**) The rat gambling task (RGT) and risky decision task (RDT) probe uncertainty-based decision-making varying in pellet (P) quantity, probability and punishment during the sessions; ‘safe’ choices are in green, whilst ‘risky’ choices are in red. (**C**) The attentional set-shifting task (ASST) examines set-shifting ability between changing reward-specific stimuli of odours or textures across trials. (**D**) The 5-choice serial reaction time task (5-CSRTT) assesses attention of responses to the light stimulus spatially, with correct nose-poke selection receiving a reward. The diagrams are adapted from Bizon et al.,^20^ Callahan and Terry,^21^ and Winstanley and Floresco.^22^

In particular, pairing behavioural paradigms that task specific cognitive elements with mPFC subregion lesions and electrophysiology recordings have substantially implicated the mPFC’s heterogeneous role in complex executive functions, including working memory, decision-making, cognitive flexibility and attention (Fig. 2). Either a radial arm maze or T-maze can assess working memory, with both task variants requiring a delay between trials and the animal remembering each reward location. T-mazes are further widely deployed to assess novel adaptive learning^23^ and demonstrate plasticity in how neuronal projections from the hippocampus, either directly or indirectly to the PFC, referred to as the hippocampal–prefrontal cortex circuit, play a critical role in cognitive and emotional regulation and memory consolidation.^24^ Decision-making involving uncertainty can be probed by either the rat gambling task or risky decision task. In the former task, rats choose between four light stimuli by nose-poking holes that vary in pellet number, probability and punishing time-out periods, whilst in the latter task rats choose between two levers (safe or risky) that deliver either one reward pellet or four reward pellets with a foot shock at increasing probability over the session. Cognitive flexibility can be examined by the attentional set-shifting task, whereby rats learn the unique odour or texture of the digging pot relevant for the buried food reward location, which must be obtained six times consecutively before the stimulus feature is changed. Alternatively, the use of touchscreen-based visual discrimination reversal learning can also assess cognitive flexibility and touchscreen-based assessments are becoming more frequently utilized now, which have previously been based upon Cambridge Neuropsychological Test Automated Battery assessments.^25^^,^^26^ Attention can be assessed by the 5-choice serial reaction time task (5-CSRTT), which requires the animal to nose-poke in the correct light stimulus hole only when it flashes in order to receive a reward at the food magazine^10^ (Fig. 2).

### The mPFC in working memory

Working memory is a term definable as a system enabling short-term storage and manipulation of information (timescale of seconds to minutes) needed to perform various complex cognitive tasks.^27^ The mPFC has been substantially implicated in working memory processes. Specifically, early rodent studies involving tasks such as the delayed response and (non-)matching-to-sample has showed significant deficits for spatial or visual object information after mPFC lesions.^28^^,^^29^ However, these predominately plPFC (mPFC sub-region) lesions only appear to impair the ability to transiently alternate spatially after a delay in a T-maze or radial-arm maze and instead may reflect ancillary PFC functions or motor-mediating strategies.^30^^,^^31^ Moreover, other studies utilizing radial arm maze paradigms suggest that the rodent plPFC/ilPFC may be more important for specific ‘working-with-memory’ processes, by manipulating previously acquired information needed rather than temporarily storing across a time delay.^32^^,^^33^

Electrophysiological evidence in rodents recording multiple single cells using tetrodes has equally resulted in a mixed picture of changes in neuronal ensemble firing rates and patterns in that only a few mPFC neurons discharge spatial working memory transient signals differently during delay periods of maze tasks, with some cell assemblies predicting spatial locations.^34^^,^^35^ However, it appears that both spatial and outcome-related neuronal activity is important, as Yang and Mailman showed using a spatial working memory T-maze task that single mPFC neurons varied spatially task-related information, whereas at the population level the primary neuronal representation was outcome-related so as to ensure effective task performance.^36^

Non-human primate studies have instead largely suggested the dlPFC is essential for working memory, as early influential lesion studies exhibited evidence that dlPFC damage, particularly involving the principal sulcus (glossary, Box 1), resulted in profound impairments in maintaining spatial information during similar delay-response or alternation tasks.^37–39^ Numerous electrophysiological unit recordings in primates have also shown persistent delay-period neuronal activity from the dlPFC or ventrolateral PFC during spatial or visual object tasks respectively, which were thought to indicate temporary information storage.^40–43^ Although more recent data displayed both spatial and non-spatial elements are equally processed across the whole lPFC, which still remains debateable today concerning the mechanisms underpinning delay period activity.^44–46^

Perhaps unsurprisingly, work investigating the PFC-functional associations in humans appears to be closer to what is described in primates rather than rodents. Human lesion studies have reinforced the notion that the lPFC is necessary for working memory function.^47^^,^^48^ Moreover, early neuroimaging studies involving PET (glossary, Box 1) and fMRI have equally activated the human lPFC in processing working memory information during numerous spatial, non-spatial and *n*-back tasks.^49^^,^^50^ However, some human studies still agree with aspects of rodent work by suggesting that the human PFC may have a greater role in cognitive control processes rather than simply maintaining representations.^48^^,^^51^

### The mPFC in decision-making

The executive function of decision-making is the ability to select an advantageous response from an array of possible options.^52^ Although possibly understudied, the reinforcer devaluation task (glossary, Box 1) paired with lesions has interestingly implicated the orbitofrontal cortex (OFC) in specifically adjusting to reward value changes that has been further localized within the macaque.^53^^,^^54^ The OFC itself has previously been clarified as one of the key regions involved in olfactory discrimination with taste reward, which suggests that olfactory sensory stimuli may be a key confounding factor for rodents when performing behavioural tasks and thus may require further assessment in future studies.^55^ Regardless, further neurophysiological recordings have still demonstrated that the OFC may update and compare values of choice outcomes, whilst the ACC seemingly evaluates and translates option values into future plans of action.^56^^,^^57^

Lesion-based animal models further implicated the mPFC in processing values with the delay-discounting phenomenon, which showed impulsive choice-related behaviour through the willingness to acquire immediately available smaller rewards instead of waiting for larger ones.^10^ In particular, temporary inactivation of ilPFC, plPFC and MO subregions increased impulsive choice, whilst increased preference for large delayed rewards/no change increased risky choice selection following MO disruption.^58–60^ However, a more recent study has verified that multiple rodent mPFC subregions, including the MO and ACC, co-operate to conduct value-based decision-making by displaying specialization with functional overlap.^61^ Indeed, further inactivation studies have implicated the ilPFC/plPFC is necessary for optimal uncertainty-based decision-making during changing risk-reward contingencies.^62^^,^^63^ Interestingly, both the OFC and mPFC have recently demonstrated distinct contributions during a rodent risk-based decision-making task with the OFC encoding overall choice value for learning and strategy updating, whilst the mPFC appears to execute strategy and monitor reward outcome.^64^

The deficits highlighted in animal work concerning the reinforcer devaluation task has seemingly resembled aspects of human experiments by demonstrating that neither monkeys nor humans appear to factor in expected outcome values after vmPFC/MO damage.^65^ Neuroimaging studies have additionally adapted the task for use in humans and have equally suggested a network involving the MO and ACC represents differing reward value elements when making a choice.^66^^,^^67^ Yet, animal–human similarities are not apparent for delay-discounting due to the mPFC/MO and lPFC being implemented, which may be attributed to the task delay duration between humans (hypothetical seconds to months) and animals (seconds to minutes) varying.^68^^,^^69^ Although, vmPFC damage has typically exhibited suboptimal uncertainty-based decision-making whilst performing the Iowa Gambling Task (glossary, Box 1), this association has later not been made clear with the dlPFC also contributing to measurable executive dysfunction.^70^^,^^71^ Regardless, fMRI studies in healthy young adults have replicated original findings of vmPFC involvement in coupling working memory and emotional state representations together whilst performing the Iowa Gambling Task, which may represent the disparities of neuroimaging only detecting activity associations whilst lesion-work exemplifies functions through impaired performances.^72^ Collectively, these findings in humans mainly parallel the animal studies by highlighting a complex collection of OFC, ACC and additionally dlPFC regions but not strictly the mPFC as a significant contributor.

### The mPFC in cognitive flexibility

Cognitive flexibility (also known as behavioural flexibility) is a key executive function mediated by the PFC and can be defined as the ability to adapt behaviour with changing environmental contingencies.^73^ Two of the most extensively studied components within this area subserved by distinct PFC subregions include attentional set-shifting and reversal learning. Set-shifting consists of attentional response shifts to differing stimuli across diverse dimensions with changing reinforcement value, whereas reversal learning can refer to modifying responses with altered reinforcement contingencies after a discriminatory stimulus rule was acquired.^74^^,^^75^ Extensive evidence demonstrates that set shifting performance is critically dependent on the dlPFC in primates, or the rodent homologue, the mPFC.^76^^,^^77^

Set-shifting ability is typically assessed in the clinic by utilizing the Wisconsin Card Sorting Test (WCST), which ultimately involves a shifting response by the individual switching their attention between variable perceptual categories based upon changing card-sort rules.^78^ Although, successful performance of the WCST has previously been suggested to engage additional executive functions such as working memory by the dlPFC in monkeys, thereby similarly paralleling the limitations plaguing the Iowa Gambling Task during decision-making.^79^ The WCST does seemingly possess though the ability to compare monkeys and humans performing the exact same task including extra-dimensional, intra-dimensional and reversal learning stages, thereby enabling clearer translation and understanding of findings across species.^10^ A WCST analogue initially used in marmosets showed that lPFC excitotoxic lesions generated deficits only during extra-dimensional shifts, whilst OFC lesions produced perseverative deficits with stimulus reversal.^80^^,^^81^

Set-shifting procedures have additionally been modified for testing of structure–function relationships in rodents with substantial similarity to non-human primates. For example, Birrell and Brown designed a seminal set-shifting task for rats, which included extra-dimensional, intra-dimensional and reversal learning stages akin to the monkey version. However, findings in rats were different from those in monkeys because mPFC lesions demonstrated similarly impaired extra-dimensional set-shifting, whilst ACC lesions impaired intra-dimensional set-shifting.^82^^,^^83^ These dissociable set-shifting effects in rats have later been replicated in mice utilizing odour discrimination tasks.^84^ Hence, these results provide evidence of substantial functional homology across species, apart from the apparent mPFC and lPFC differences.

Human studies assessing cognitive flexibility processes have largely implicated similar PFC subregions compared to the animal literature, thus emphasizing the suitable translatability of the models. Specifically, individuals largely with dlPFC and mPFC damage have reported difficulties performing set-shifting during the WCST, such as inabilities to switch to a new rule as well as random and perseverative errors.^85–87^ Two meta-analyses of neuroimaging studies have suggested a broad network of regions including the lPFC and ACC activate during successful set-shifting, yet a magnetoencephalography (glossary, Box 1) study later found the ACC to possibly have a more consistent role in feedback processing or error monitoring.^88–90^ Taken together, OFC damage impairs reversal learning, whilst lateral or medial PFC damage impairs extra-dimensional set-shifting, suggesting a functional dissociation exists between these regions.^77^^,^^91^

Lesion studies of the mPFC have also been shown to play a role in impaired reversal learning, specifically when rodents are presented with complex images using touchscreens.^92^^,^^93^ Age-related alterations in both the architecture and molecular composition of the PFC are known to contribute to cognitive decline seen in healthy aged animals.^94^^,^^95^ Consistent with this, Houlton et al.^96^ revealed an age-related decline in visual discrimination reversal learning in aged animals. This finding is supported by human, primate and rodent reversal studies that have reported cognitive slowing in aged cohorts using other cognitive assessments.^94^^,^^95^^,^^97^^,^^98^ Moreover, this age-related cognitive slowing is not only applicable to behavioural flexibility but other cognitive domains, such as spatial memory, attention and working memory.^98^^,^^99^ Similarly, lesions targeting deeper regions of the PFC, such as the OFC, have also been shown to selectively impair reversal learning on visual-cue and set-shifting tasks in rodents.^76^

### The mPFC in attention

As with other domains of executive function, attention is a complex cognitive process with various components that seem to depend on differing PFC subregions. Attention enables the brain to allocate sensory resources efficiently for the immediate goal whilst ignoring alternative irrelevant inputs.^100^ This ability particularly integrates multiple components, which divide into several distinct forms including selective, divided and sustained attention as well as attentional control of task performance. The rat-based 5-CSRTT has become a widely implemented method to assess an animal’s ability to maintain attention to unpredictable visual stimuli across five different spatial locations. Muir et al.^101^ initially demonstrated using the 5-CSRTT that mPFC lesion including the ACC and plPFC led to choice accuracy reduction, slower/premature responses and increased perseverative responding. Subsequent lesions precisely limited to the rostral ACC area have caused deficient response accuracy as opposed to previous more caudal ACC lesions.^102^^,^^103^ Comparatively, plPFC lesions led to greater perseverative responses and ventral ilPFC lesions only appeared to increase premature responding.^102–104^ Two temporary inactivation studies have since confirmed the prior findings by suggesting that dmPFC may mediate attentional function, whilst the ilPFC regulates inhibitory actions.^105^^,^^106^ Electrophysiological evidence suggests the plPFC and ACC might evoke preparatory attention with the ilPFC mainly controlling impulsive actions.^107^^,^^108^ A recently developed rodent touchscreen version has also suggested the plPFC detects and discriminates attentional stimuli with the ACC processing inappropriate responses.^109^^,^^110^ These results collectively demonstrate that varying attentional components need disparate rodent PFC areas, with maximal performance on the 5-CSRTT requiring the regions to interact together.

The apparent agreement of findings from rodents to humans is perhaps undeniable as the 5-CSRTT was initially developed by Carli et al.^111^ based upon Leonard’s 5-choice serial reaction task, which assesses sustained attention in human subjects. The 5-CSRTT also appears to possess some analogies to the continuous performance task (glossary, Box 1), with the later developed 5-choice continuous performance test mimicking human paradigms even closer by including inhibitory response non-target trials.^112^ An adapted 5-CSRTT in humans has demonstrated superomedial frontal lesions prolong reaction time, whereas lateral frontal lesions produced more errors over longer inter-stimulus intervals.^113^ These medially related findings seemingly correlate to rodent findings, yet they do additionally include lateral aspects. Further human lesion-based work implicates various types of attention such as endogenous visual/auditory, preparatory and spatial, which are modulated by lPFC regions.^114–117^ Despite, there still appearing to be disparate findings regarding mPFC and lPFC regions between rodents and primates as a recurring theme; this may in fact reflect the PFC structure–function behavioural field.

## Disparities and similarities of mPFC findings between rodents and primates

It has been debated over several years whether rodent mPFC studies are relevant to define human dlPFC functions, whilst others have suggested that rodent mPFC might better represent the ACC.^118^ In addition, complementary functions rather than structures with the rodent mPFC has seemingly been emphasized by prior executive function paradigms.^119^^,^^120^ Moreover, rodent OFC has previously been omitted from a proposed orbital network due to hypothalamus and periaqueductal grey connections found only in rats, even though it has mediodorsal nucleus of the thalamus projections thereby challenging Rose and Woolsey’s original PFC definition.^121^ Therefore, the term ‘prefrontal’ has remained consistently ambiguous in rodent studies. Perhaps, this may instead reflect an inadequate consensus on the anatomic nomenclature used to describe PFC subregions and how they translate across species, as clear differences in term usage and research focus between rodent and primate PFC studies has recently been established (Table 1). Future studies would therefore benefit from reporting stricter standards of anatomic terms; otherwise, cross-species comparisons could be considerably more difficult.^3^^,^^6^

Animal behavioural tasks of cognition adapted from human versions have enabled detailed investigation of brain areas by utilizing naturalistic paradigms. However, limitations arise if factors are not controlled such as food restriction, lack of motivation, susceptibility to stress and malaise or sense/locomotor impairments, which may be apparent for the prior studies analysing executive functions. Before animals perform behavioural tasks, it may therefore be necessary to ensure that they critically assess the cognitive function under investigation precisely, as exemplified by modified T-maze or operant procedures developed for working memory or cognitive flexibility respectively.^122^^,^^123^ Regardless, animal models have continued to display great promise for teasing apart mPFC-related cognition, which has also been investigated within specific rodent models of ageing and dementia-associated disorders.

## Rodent models of ageing and dementia-associated disorders involving the mPFC

In man, there is a clear deterioration in cognitive function during normal ageing, often with an observable reduction in information processing speed, which is not dependent on executive functioning.^124^^,^^125^ Such effects have implicated the mPFC as emphasized by an mPFC/ACC-linked network showing the greatest hypometabolic activity correlated to declining cognitive function.^126^ Research involving animal models of ageing has specifically demonstrated similar age-related difficulties due to changes affecting the mPFC and executive function across the lifespan. In particular, aged rodents show delay-dependent inabilities compared to younger animals whilst performing a variety of working memory paradigms. These likely task the mPFC and involve delayed alternation, radial arm and delayed match-to-sample water mazes.^127–129^ Several studies also show that aged rats exhibit declined ability to adapt their responses within extra-dimensional set-shifting and olfactory reversal learning tasks compared to younger animals.^130^^,^^131^ A similar age-related decline in set-shifting function has also been reported in mice, which is shown to be linked to decreased trophic factor signalling and in particular, brain-derived neurotrophic factor signalling.^96^ Whilst others have suggested only modest attentional impairments possibly due to low 5-CSRTT sensitivity and deficits affecting cost-accounting and reward magnitude for uncertainty-based decision-making within aged rodents.^132–134^ Executive functions also appear to interact as evidenced by functional changes in working memory and cognitive flexibility both affecting delay-discounting decision-making processes.^135^

Studies utilizing rodent models have thus not only suggested that mPFC cognitive functioning is substantially affected by ageing in seemingly complex ways but have additionally aided in elucidating the underlying pathophysiology of ageing-associated neurological disorders that are susceptible to dementia. Specifically, authors demonstrated with a photothrombosis model (glossary, Box 1) of focal stroke localized to the mPFC, an inability to discriminate novelty four weeks later in post-ischaemic stroke (PIS) mice during an object location recognition task (glossary, Box 1), which suggests delayed-onset spatial memory impairment after mPFC stroke.^136^ To ascertain an electrophysiological correlate, Hillman et al.^137^ reported a loss of PFC–hippocampal coherence in the theta band range between 2–4 weeks PIS, which corresponds with when the delayed-onset spatial memory was observed. Interestingly, however, they report a change in the beta band oscillations in the PFC that proceeds the onset of spatial memory impairment, indicating a plausible electrophysiological biomarker that could indicate if someone is likely to develop delayed-onset memory impairment.^137^

Further studies utilizing a rodent model of acute mPFC ischaemic stroke through bilateral endothelin-1 (glossary, Box 1) injections have displayed anxiogenic responses and a range of selective PIS executive dysfunction including impaired cognitive flexibility as extra-dimensional set-shifting, ability to set-shift between diverse reward cues and possibly defective memory-linked object recognition (depending upon PIS experimental timeframe or object types used potentially) relative to sham animals.^138–141^ Moreover, a recent study utilizing operant touchscreen chambers highlights significant spatial working memory impairments within a PIS rodent model targeting the bilateral frontal cortex, which also interestingly reported a positive association between white matter (WM) reactive astrogliosis and cognitive impairment.^142^ Whilst a study using a bilateral common carotid artery occlusion rat model of vascular dementia (VaD) has also assessed attentional set-shifting ability and demonstrated that these rats were slower learning only the ACC-dependent intra-dimensional task compared to controls, thereby implicating the potential neural substrate underlying similar impairments within VaD patients.^143^ Accumulating evidence indicates that pathological disturbances in mPFC function are further related to neurodegenerative disorders, as Alzheimer’s disease, Huntington’s disease and Parkinson’s disease rodent model studies utilizing amyloid-β (glossary, Box 1) peptide injection, transgenic manipulation and 6-hydroxydopamine lesions, respectively, have exhibited deficits performing mPFC-dependent working memory tasks.^144–146^ Animal models have thus continued to enable crucial and seemingly similar aspects of mPFC-associated cognition to be distinguished across disorders involving dementia, yet investigations into additional unique elements concerning mPFC networks with connections to other brain regions has recently expanded. However, in order to validate the animal findings, consensus groups have been highlighting that parallel preclinical and clinical longitudinal studies need to be established, which would allow one to identify and validate biomarkers and determine when to start treatments and which intervention to use.^147^

## The mPFC in cognitive processes and connectivity-based research

Extensive lesion work has previously suggested mPFC dissociable executive functions are limited to precise anatomic subregions in various disorders (Table 1). However, the mPFC specifically represents a heteromodal region (glossary, Box 1) with connections to other heteromodal brain areas, which enable key interactions necessary for optimal cognition.^148^ Various disconnection, electrophysiological and recently optogenetic (glossary, Box 1) rodent studies have demonstrated mPFC synchrony with sub-cortical and limbic structures, such as: ventral hippocampus for working memory, amygdala/dorsomedial striatum for decision-making, dorsomedial thalamus/ventromedial striatum for cognitive flexibility and sub-thalamic nucleus for attentional processes.^149–156^ Moreover, lesion effects are not always limited to circumscribed locations due to diaschisis (glossary, Box 1) affecting remote connected sites and the damage commonly overlapping nearby subregions, therefore, greater understanding of mPFC structure–function relationships requires a cortical network-based approach.^157^ Such a network-based approach with measures of connectivity may sufficiently aid in resolving disparities between previously highlighted rodent and primate study findings. A neuroimaging approach has recently revealed homologous mPFC activation in macaques and humans during decision-making.^158^ This approach may therefore prove useful for comparing rodent areas, with cross-species cortical–striatal connectivity patterns already being reported.^159^

Further studies in humans using a range of neuroimaging techniques have suggested specific networks are key for cognitive domains. In particular, fMRI and voxel-based lesion-symptom mapping (glossary, Box 1) of WM fibre tracts have interestingly identified a widespread fronto-parietal network (glossary, Box 1), which is sensitive to working memory tasks and contained a restricted core network of posterior mPFC and caudal lPFC regions.^160^^,^^161^ Functions in additional types of memory have recently been determined, since a network involving the dmPFC has suggested a causal role supporting perceptual memory, whilst hippocampal–mPFC connections have emerged for episodic autobiographical memory and prospective-guided memory for decision-making.^162–164^ Assessing decision-making under certain or uncertain conditions appears to respectively recruit a network containing either the vmPFC or bilateral PFC; thereby suggesting particular networks are critical for precise roles even within the same cognitive domain.^165^ Alternatively, applying a voxel-based lesion symptom mapping approach has revealed the necessity of a vmPFC-containing network for value-based decision-making, with set-shifting further requiring a rostral ACC control network.^166^

Large-scale interconnectivity networks oversee a range of complex cognitive roles. The default mode network (DMN) (glossary, Box 1) in particular has implicated the mPFC as one of its central nodes, as reviewed here. Unexpectedly it was first identified in neuroimaging studies as regional signal decreases during goal-directed tasks relative to a baseline resting brain state.^167^^,^^168^ This system can be separated into three major cortical sub-divisions: vmPFC, dmPFC and posterior cingulate cortex (PCC)/medial precuneus plus the lateral parietal cortex (LPC) and entorhinal cortex. Human data investigating these subregions have suggested the DMN supports emotional processing (vmPFC), self-referential activity including mentalising/social cognition (dmPFC) and recollecting past experiences or envisioning the future (posterior DMN components).^169^^,^^170^ The thalamus and basal forebrain subcortical structures have recently been included within a more comprehensive DMN model as important functional elements.^171^ Further studies have suggested that the DMN has more refined roles in path integration/navigation, orienting in space, time and person as well as mind wandering.^172–174^ However, the observation of resting-state activity transcending beyond levels of consciousness may question the latter association with the DMN.^170^

## mPFC connectivity in ageing and disease

Rodent models of ageing and disease along with large-scale brain connectivity neuroimaging studies have equally emphasized that the mPFC has a diverse role in cognition. Therefore, combining these areas together may provide insight into the aberrant mPFC structural and FC changes underlying compromised neuronal function in ageing and dementia-associated neurological disorders. A large number of cross-sectional rs-fMRI based studies, which measure spontaneous neural processing through blood oxygenation level-dependent signals in distinct brain regions without tasks, have reported network disturbances within cognitively dysfunctional individuals in recent years.^175^^,^^176^ Yet, little consensus has clearly been established due to several inconsistencies remaining in the literature, which result from small sample sizes and a plethora of methodological differences.^143^ However, can these studies help us determine how disparate connections involving mPFC circuitry may differ across ageing and dementias? If so, they may reveal disease-specific neural substrates or pathological processes and ultimately provide viable biomarkers as well as refined targets for implementing therapeutic treatments.

We accordingly hypothesized that some functional mPFC connectivity differences will be apparent with ageing at an early stage and between various disorders reflected in a number of features in executive dysfunction (Table 2). We surmised that this would be particularly prevalent within specific mPFC-linked networks such as the DMN, which has previously been identified as disturbed during pathological states such as Alzheimer’s disease. In view of the overlap between cerebrovascular disease and Alzheimer’s disease pathologies,^177–181^ it would be of interest to additionally delineate changes in mPFC FC, that are driven by vascular mechanisms and establish age as a key factor in the detrimental effects upon cognition.^182^ Thus, deciphering if mPFC-specific rs-fMRI FC brain changes related to cognitive dysfunction occur in ageing and across a range of cognitive impairment and dementing disorders (Table 2). These findings may additionally demonstrate unique disease-specific patterns of mPFC FC alterations within specific large-scale networks, which appear to consistently feature the DMN, whilst detrimental connectivity alterations are associated with cognitive impairments independently from structural pathological aberrations such as grey matter (GM) atrophy but may arise as a result of WM changes.

**Table 2:** The prefrontal cortex and executive dysfunction in ageing-related neurocognitive disorders

Group	Disorder(s)/disease(s)	Executive dysfunction features^a^
Prodromal syndromes	Mild cognitive impairment	Working memory
Alzheimer syndrome	Alzheimer’s dementia Mixed dementias	Frontal phenotypes; working memory, cognitive flexibility (set-shifting), inhibition (self-control)
Synucleinopathies	Dementia with Lewy bodies Parkinson’s disease Multiple system atrophy	Verbal reasoning, problem-solving, ability to maintain sustained attention
Tauopathies	Frontotemporal dementias Corticobasal degeneration Progressive supranuclear palsy	Working memory, inhibition (self-control), cognitive flexibility
Vascular cognitive impairment (VCI)	Mild/Severe VCI Vascular dementia Multi-infarct dementia Subcortical vascular dementia Post-stroke dementia	Working memory, planning, verbal reasoning, problem-solving, ability to maintain sustained attention, resistance to interference, multitasking
Trinucleotide repeat disorders	Huntington’s disease	Verbal reasoning, fluency, problem solving

a Executive function may include several other domains and it is dependent on information processing speed, which can be affected in several disorders, particularly those exhibiting disruption of the subcortical white matter.

To ensure our findings were specifically focused on mPFC FC, we concentrated on reviewing relevant articles on rs-fMRI from the PubMed and Scopus databases (January 2000 to June 2020) that revealed significant differences between ageing and dementia-associated disorders in terms of mean connectivity to brain regions involving the mPFC. The current data present 41 published studies totalling 2473 subjects with an average of 60 per study (Table 3). The most relevant groups were aged (range 60–77 years) individuals, mild cognitive impairment (MCI), vascular cognitive impairment (VCI), Alzheimer’s disease dementia, Parkinson’s disease and frontotemporal dementia (FTD) patients. Twenty-nine studies (70.7%) investigated rs-fMRI FC associations with other domains, cognition being the most common, whilst other areas of importance were brain atrophy (glossary, Box 1) or GM volume and structural connectivity (glossary, Box 1). Executive dysfunction has also been suggested as a predictor for VCI in post-stroke cases.^179^^,^^183^^,^^184^ The frontal lobe is particularly vulnerable to vascular-based pathology and disruption of the striato-pallido-thalamo-cortical circuit is common in VCI and VaD, which may result from subcortical lesions affecting connectivity between the PFC regions including the dlPFC, mPFC and thalamic nuclei. Studies assessing the relationship between the location of lacunar infarcts and cognitive domains reported that impaired information processing speed is explained by disruption of circuits between the anterior thalamic radiation (and the forceps minor) or the anteromedial thalamic nucleus and the prefrontal cortex (mPFC).^185^^,^^186^

**Table 3:** Summarized cohorts and methodology features of rs-fMRI in various studies

Disorder	Ageing	MCI/AD	svMCI/PIS	PD/APDs	FTD
Number of studies	10	12	10	7	2
Mean total group (*N*)	74.3	62.3	42.2	66.7	47.0
Mean total female (%)	48.3	48.6	39.0	46.3	43.6
Mean total age (years)	57.3	69.3	62.7	66.5	64.4
Scanners used	3 T S, 1.5 T S, 1.5 T GE, 3 T	1.5 T GM, 3 T S, 3 T P, 2 T* S, 3 T GE, 1.5 T GE, 1.5 T S	3 T P, 3 T S, 3 T GE, 1.5 T S	3 T S, 1.5 T GE, 3 T, 3 T P, 1.5 T S	3 T P
Methods used	VB, ICA, SB, ICA/SB	SB, ICA, VB, ICA/SB, VB/GT	SB, GT, VB, ICA, ICA/SB/GT, ICA/SB, ICA/VB	SB, ICA/SB	ICA, SB/VB

Studies were selected here for each disorder category by only including subjects aged over 50 years old and those withmedial prefrontal cortex functional connectivity differences between aged or disorder participants and age-matched cognitively unimpaired or healthy controls. A full, detailed version of the cohort features and methodologies used for each study as well as the regions and network(s) investigated is provided as Supplementary Table 1 within the Supplementary material.

AD, Alzheimer’s disease; APDs, atypical Parkinsonian disorders; FTD, frontotemporal dementia; GE, General Electrics; GT, graph theory; ICA, independent component analysis; MCI, mild cognitive impairment; PD, Parkinson’s disease; P, Philips; PIS, post-ischaemic stroke; SB, seed-based; S, Siemens; svMCI; subcortical vascular mild cognitive impairment; T, Tesla; VB, voxel-based.

### Healthy ageing

There is a consistent decrease in mPFC–PCC FC in healthy aged individuals (Supplementary Table 1). Although this decreased trend was also apparent between the mPFC and parietal cortices, the exact mPFC subregion contributing to the connectivity change interestingly differed for both connections. As Vidal-Piñeiro et al. and Andrews-Hanna et al. reported the mPFC, whereas the other two studies suggested more precise subregions of dmPFC or ACC are affected.^187–190^ These slight discrepancies may reflect inconsistencies in mPFC terminologies used (thus carrying over from animal work) along with the precision of the scanner to detect the signal rather than data analysis disparities, as almost the exact same independent component analysis (ICA) (glossary, Box 1) and seed-based (glossary, Box 1) approaches were implemented.^3^ Previous pioneering studies have also interestingly shown that FC reductions between anterior mPFC and posterior DMN connections associated with decreased structural measures of WM and GM integrity in the cingulum tract and distributed across the brain within areas of high age vulnerability.^187^^,^^190^ This implicates that both functional and structural alterations during ageing may impact upon one another to accelerate the subsequent decline in cognitive performance.

Furthermore, the PCC-insula reduced FC association has been positively correlated to cognitive tests including those for executive function, along with decreased FC with ageing in ACC connections to the insula as part of the salience network (glossary, Box 1).^189^^,^^191^ The former connection has been disputed though by also showing the converse relationship of stronger FC with age, which may be due to parcellating the DMN into distinct dorsal and ventral PCC subsystems, rather than assessing the PCC FC in its entirety.^188^ Alternatively, such an increased activity trend within the PFC may instead represent compensation rather than methodological effects. Some studies have hypothesized this could reflect posterior-to-anterior shift or suggested that it is rather reduced efficiency in response to cognitive impairment during healthy ageing.^192^^,^^193^ Yet, the PCC has also displayed similarities by linking decreased FC with ageing to the vmPFC.^188^^,^^194^ Therefore, together these observations indicate distinct cognitively important mPFC subregion FC changes with most suggesting a reduction with increasing age.

### Prodromal Alzheimer’s disease

Although the prior section focused upon healthy ageing, with a study displaying network alterations without signs of Alzheimer’s disease pathogenesis, others have investigated FC changes in those cognitively normal but with toxic Alzheimer’s disease hallmarks such as high amyloid-β burden.^187^ Some studies have implicated decreased FC between mPFC/ACC and hippocampal regions in these individuals, therefore, indicating a preclinical stage of Alzheimer’s disease. It was not clear from these studies whether specific regions of the hippocampus i.e. anterior versus posterior are affected but it is likely that hippocampal formation as well as the parahippocampal gyrus is involved. However, they still suggest differing associations, with reduced LPC, PCC and hippocampal FCs being shown in only one study, which may be due to disparate seed regions utilized.^195^^,^^196^ Potential issues introducing bias by specifically choosing the seed regions to investigate was further demonstrated in two studies assessing the impact of only carrying the apolipoprotein E ε4 (*APOE4*) (glossary, Box 1) Alzheimer’s disease risk factor allele, which also suggested altered connectivity in cognitively key mPFC/ACC and hippocampus areas. Yet, variances in FC direction using a precuneus seed region were also found; thus, more similar and comparable methods in future studies for this area would likely be useful.^197^^,^^198^

### Mild cognitive impairment

Considerable mPFC FC trends in individuals who have amnestic MCI are also apparent. In particular, weakened FC in MCI between the hippocampal formation and mPFC.^199^^,^^200^ Whilst another study found almost complete loss of mean hippocampal–mPFC signal in MCI/mild dementia patients.^201^ Hence, these observations collectively correspond since Alzheimer’s disease tau pathology (glossary, Box 1) initially accumulates in the entorhinal cortex/hippocampus and may intriguingly reflect a prion-like tau spread from the medial temporal lobes (MTLs) to the mPFC.^201–203^ Indeed, some of these PFC/hippocampal FC changes may plausibly reflect alternative mechanisms driven in part by secondary factors, such as changes in cholinergic innervation or structural damage to the fornix, since the former in particular provides innervation to both the PFC and hippocampus.^204^ Moreover, reduced FC between the mPFC and PCC has been observed in MCI. This was found without structural PCC GM atrophy in Gili et al. and coincides with PET studies showing PCC metabolic decline in early Alzheimer’s disease. Thereby possibly representing mPFC/hippocampal structural GM atrophy that alters functional circuits and may even precede PCC structural aberrations, which thus lead to worsening cognitive deterioration through disrupting the DMN’s functional circuits.^205^^,^^206^

In contrast, greater FC between the mPFC and PCC or inferior parietal lobule in MCI compared to ageing controls has also been reported.^199^^,^^207–210^ These findings may thus represent network compensation, as previous studies have suggested PFC FC increases during short-term memory tasks so as to temporarily maintain cognitive functioning.^199^^,^^209^^,^^210^ However, Gardini et al.^208^ interpreted this increased FC as a maladaptive response to initial neuronal loss with detrimental lower levels of DMN deactivation at rest. This is different from previous findings by showing increased mPFC–hippocampal FC negatively correlates with semantic memory performance; yet, these inconsistent findings may represent varying progression phases and clinical heterogeneity among MCI subjects.^208^^,^^211^

### Alzheimer’s disease

Once patients have progressed from MCI to a more advanced clinical state of Alzheimer’s disease dementia, a clear trend in the decline of mPFC connectivity emerges. There is decreased DMN FC between the mPFC and parietal cortices or PCC compared to healthy ageing controls.^197^^,^^201^^,^^205^^,^^212^^,^^213^ Interestingly, no mPFC–hippocampal connections are reported unlike the MCI cohorts displaying an FC reduction.^199^^,^^200^ This finding has been verified in studies only showing this connection in healthy controls or MCI patients, whilst Alzheimer’s disease patients across cohorts have possessed the greatest structural measure of MTL GM atrophy, which may have advanced to the stage of complete disconnection from the mPFC.^201^^,^^205^ We have previously reported that MTL atrophy even in Alzheimer’s disease could be explained by a purely vascular mechanism independent of the presence of Alzheimer type of pathology.^179^^,^^214^^,^^215^ The mPFC–PCC connection was also shown to possess more severely declined FC in Alzheimer’s disease patients compared to MCI, yet another study implicated the ACC rather than the mPFC is affected in this connection.^205^^,^^212^ These apparent discrepancies in detecting precise mPFC subregions could similarly parallel previous ageing findings with scanner and terminology inaccuracies. Yet, both Alzheimer’s disease studies in particular had relatively small sample sizes, average of 12 participants per cohort, meaning that significant differences are possibly not detected with substantial statistical power. Vipin et al.^213^ have further suggested region-specific changes of increased intra-DMN mPFC-parietal FC within Alzheimer’s disease and MCI patients with significant cerebrovascular brain pathology; thus, demonstrating that vascular aberrations may further influence deleterious mPFC network-based degeneration.

### Subcortical vascular mild cognitive impairment

Detrimental vascular modulations of FC within mPFC networks have not only been reported in MCI or Alzheimer’s disease, but also in those at an earlier prodromal state for VaD or VCI with subcortical vascular mild cognitive impairment (svMCI), which is predominantly characterized by executive dysfunction. Indeed, svMCI subjects exhibit significant declines in numerous DMN-associated regions compared to controls, which may result structurally from subcortical WM lesions that directly and indirectly impair fibre tracts essential for transmitting cerebral FCs. These regions specifically include the PCC/precuneus, mPFC, ACC, hippocampus, parietal cortices and superior frontal gyrus/middle frontal gyrus.^216–218^ Nevertheless, the exact mPFC-related connections are perhaps not completely deducible since minimal clinical variable associations were obtained, possibly due to methodological divergences created by biased hypothesis-driven analytical approaches selecting contrasting seed regions of PCC or thalamus regions.^216^^,^^218^ Whilst another study perhaps preferred a more reliable data-driven graph theory (glossary, Box 1) approach based upon topological attributes and modularity structure, yet it contrasted findings by suggesting increased within-module/sub-network degree of mPFC, left insula and cuneus regions within svMCI subjects.^219^ Disparities in findings may additionally stem from the influence of medications upon brain activity, along with subject heterogeneity since very small lesions were disparately distributed throughout the brain and two studies reported slight volume atrophy potentially affecting some FC results.^216^^,^^217^

### Post-ischaemic stroke

Approximately 30% of elderly stroke survivors develop delayed dementia (known as post-stroke dementia), with most cases closely resembling criteria for VaD diagnosis.^220^^,^^221^ Current studies suggest analogous trends to svMCI for this increasingly important PIS population in terms of variable mPFC associations. Several studies assessing predominantly first-time ischaemic stroke individuals have collectively exhibited elevated mPFC and hippocampal FC, which may reflect compensatory processes as a result of structural damage and deterioration of extra-frontal regions.^222–225^ Although raised FC through connections with the precuneus was further implicated, either the mPFC or ACC contrastingly mediated this connection, potentially due to dissimilar graph theory or ICA assumptions of statistical independence for identified components.^222^^,^^225^^,^^226^

However, lowered mPFC/ACC-precuneus FC was conversely demonstrated by utilizing similar group ICA/region-of-interest methodologies and rather reflects the impact upon structural damage facilitating cognitive disturbances as a disconnection syndrome.^224^^,^^227^^,^^228^ Perhaps, the disparities in findings may be due to differences in timings of the rs-fMRI scans PIS being taken either acutely or sub-acutely, as this particularly varied amongst the studies. Moreover, Park et al.^224^ supported this assertion by showing that mPFC FC changes occurred longitudinally PIS with decline at one month, gradual restorations to recover cognition at three months and compensatory increases for persistent PCC/precuneus reductions at six months. Yet, another study showed increased mPFC/hippocampus FC scanned 5–10 days PIS and even demonstrated this trend at a lower intensity in cognitively impaired PIS individuals, meaning heterogeneous patient characteristics such as variable lesion sites/sizes and vascular risk factor differences (e.g. hypertension), which can confound resting-state FCs appear to be more plausible reasons.^218^^,^^222^^,^^223^ Intriguingly, another potential causal link for the cognitively impaired PIS individuals may stem from WM vascular pathology substrates such as reactive astrogliosis or clasmatodendritic changes causing end-feet retraction from microvessel and blood–brain barrier damage, which has been found to be significantly elevated within post-stroke dementia subjects at post-mortem.^229^ Such pathological changes at a prefrontal cellular level due to vascular malformations equally corroborates with prior evidence of highly selective dlPFC pyramidal cell atrophy arising within post-stroke dementia and VaD subjects.^178^

### Parkinson’s disease

Parkinson’s disease as a neurodegenerative disorder is typically characterized by progressive motor dysfunction, but patients also show cognitive decline with executive deficits, memory impairment and often dementia in advanced stages.^230^^,^^231^ The cognitive deterioration is seemingly evident in rs-fMRI, as revealed by diminished FC within recurrently susceptible DMN-linked regions.^232–235^ Specifically, stronger DMN anterior–posterior circuit connectivity amongst the mPFC, PCC, inferior parietal cortex/LPC and MTLs has been reported within controls relative to early Parkinson’s disease patients at resting-state, thereby implicating pathological mPFC circuit disruption.^232^^,^^233^

However, similar disparities in trends as demonstrated in the prior vascular studies, are prevalent within the Parkinson’s disease studies. As mPFC FC changes compared to controls did not appear in two studies, which instead only showed significant FC decreases that associated with cognitive performance or lower GM volume (as well as reduced fractional anisotropy in WM adjacent to DMN regions) structurally between the precuneus/PCC and subcortical/motor areas or medial temporal gyrus.^234^^,^^235^ Dopamine replacement therapy has been concluded to critically affect functional brain organization and thus may explain these differences in trends, yet Lucas-Jiménez still showed PCC-MTL aberrations without controlling for this levodopa equivalent dosage indicating this may be unlikely.^234^^,^^236^ Alternatively, these discrepancies may reflect variable motor symptom severity in patients, as implicated mPFC involvement had higher Hoehn and Yahr scores (glossary, Box 1), which were associated with greater cognitive deficits and thereby possibly represent weakened DMN hubs like the mPFC.^232^^,^^237^ A Parkinson-related dementia cohort study comparatively only exhibited reduced caudate-middle frontal cortex FC, indicating FC deviations specific to subcortical Parkinson’s disease pathology can arise at a more advanced stage.^238^ Neuroinflammation may also influence the FC given substantial increases in astrogliosis, microgliosis and pro-inflammatory markers were shown recently within the PFC of X-linked Dystonia-Parkinsonism patients.^239^

### Atypical Parkinsonian disorders

Despite stringent Parkinson’s disease clinical criteria, there remains a substantial misdiagnosis rate with atypical Parkinsonian disorders (APDs), such as multiple system atrophy (MSA) and progressive supranuclear palsy (PSP), even though APDs account for 10–20% of Parkinsonism subjects.^240^^,^^241^ MSA and PSP patients often manifest multiple cognitive deficits during disease progression. Recent studies have respectively investigated the underlying cognition-related FC changes in either disorder through similar seed-based rs-fMRI protocols. Both studies paralleled the dementia-associated conditions by demonstrating significantly impaired cognitive performance potentially resulting from reduced memory-linked DMN mPFC FCs after correcting for structural GM volume loss. However, distinct pathological processes may underlie each disorder since explicit cerebello-cerebral network disruptions occurred in MSA, with more typical anterior–posterior mPFC–PCC disorganization and mPFC-motor network compensatory FC increases in PSP subtypes.^242^^,^^243^ Therefore, MSA cerebellar and PSP cortical neurodegeneration may cause widespread network disconnection and DMN abnormalities before structural aberrations arise, which has been reported for MSA and corresponds to pathological tau protein post-mortem deposition within PSP being reported in the same aberrantly altered FC regions.^242–244^

### Frontotemporal dementia

The most common form of FTD is the behavioural variant, which has previously been shown to account for approximately half of all frontotemporal lobar degeneration (glossary, Box 1) disorders and along with Alzheimer’s disease (Table 2), is the most common aetiology of early-onset neurodegenerative dementia.^245^ Interestingly, an rs-fMRI study utilizing subjects with this subtype of FTD suggested reduced FC between several long-range pairs of mPFC-related components within the posterior DMN and attentional networks (Supplementary Table 1). Altered power spectra were found within the dmPFC and this region was further shown structurally to possess significantly reduced GM density, with a positive association between the anterior DMN component and affective mentalising task scores.^246^ In addition, within the temporal variant of FTD, semantic dementia, which involves GM atrophy progression from the temporal lobes to the frontal lobe thus leading to semantic memory impairments as well as social cognitive deficits over time. Bejanin et al.^247^ showed subjects had decreased FC between midline cortical regions involving the mPFC and temporal regions despite local GM atrophy. However, these FC trends were not correlated with impaired theory of mind performance.

Nonetheless, considering the apparent involvement of mPFC-dependent networks, FTD is not widely explored compared to other dementia-associated disorders. Instead, it has largely revolved around structural or apathy task-based neuroimaging records, indicating there is an obvious requirement for future research to further elucidate mPFC network changes.^248^^,^^249^

### Overall mPFC connectivity change trends across ageing and disorders

**Figure 3:**
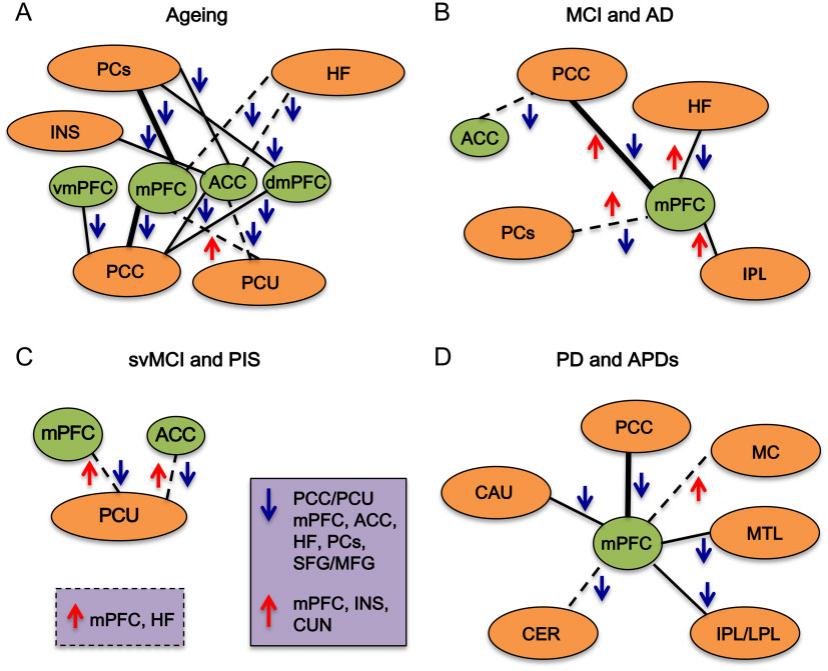
**Summary of altered mPFC FC trends across ageing and disorders.** (**A**) Pairwise FC changes (upwards red arrow indicates an increase and downwards blue arrow a decrease) in healthy (thin line), Alzheimer’s disease susceptible (dashed line) or both (thick line) aged subjects between mPFC subregions (green circles) and parietal cortices (PCs), insula (INS), hippocampal formation (HF), posterior cingulate cortex (PCC) and precuneus (PCU) brain regions (orange circles). (**B**) Pairwise FC changes in MCI (thin line), Alzheimer’s disease (dashed line) or both (thick line) between mPFC subregions and PCC, HF, PCs or inferior parietal lobule (IPL). (**C**) Pairwise FC changes in PIS (dashed line) between mPFC subregions and PCU; connectivity aberrations (purple box) in svMCI (solid outline) and PIS (dashed outline) between mPFC subregions and HF, PCC/PCU, PCs, superior frontal gyrus/middle frontal gyrus (SFG/MFG), insula (INS) and cuneus (CUN). (**D**) Pairwise FC changes in PD (thin line), APD (dashed line) or both (thick line) between the mPFC and PCC, caudate (CAU), cerebellum (CER), IPL/lateral parietal lobule (LPL), medial temporal lobe (MTL) and motor cortex (MC). The FTD study trends are not provided in order to remain succinct, as several mPFC connections were displayed across the two studies.

Collectively the mPFC possesses a range of corresponding couplings that vary across ageing and disorders in terms of directional intensity (Fig. 3). In terms of network disturbances, the DMN has particularly arisen as a recurrently affected large-scale circuit involving the mPFC across ageing-related disorders, perhaps due to its common role in memory consolidation or autobiographical processes.^182^^,^^250^ Specifically, decreased FC consistently arises between long-distance anterior and posterior subsystems, which has been confirmed by sophisticated network-based ‘effective connectivity’ (glossary, Box 1) measures in MCI, Alzheimer’s disease and even *APOE4* elderly carriers.^201^^,^^251^^,^^252^ Additional mPFC connections within networks that were found to be disturbed include the salience network within healthy ageing and FTD patients, implicating that a range of critical circuits for optimal mPFC function are affected within health and disease alike. However, each disorder has also demonstrated distinct disease-specific patterns, as ageing acts seemingly on a continuum of declining mPFC FC, which continues into MCI and then Alzheimer’s disease with progressively exaggerated decline as the individuals worsen in cognitive state. Moreover, in Alzheimer’s disease, the mPFC circuits uniquely disconnect from the hippocampus and may impact upon the symptomatic memory deterioration within individuals. Vascular aberrations show highly variable trends of FC changes that are likely dependent upon the initial locus of damage, whilst Parkinsonian and FTD disorders instead largely implicate either subcortical or frontal lobe circuits to the mPFC being affected wherein pathological processes characteristically initiate and represent the underlying clinical presentation. It was also determined as another main feature of this study that both neurodegenerative and cerebrovascular disorders significantly implicated mPFC connections with subcortical areas at resting-state, however, the specificity of these connections may perhaps be clearer to elucidate for neurodegenerative disorders such as Alzheimer’s disease due to a more characteristic deterioration occurring, which typically first begins within the hippocampus affecting memory function. Of course, this is far less clear for cerebrovascular disorders, which differ largely on an individual-to-individual basis and predominantly could affect the far-reaching WM tracts to a greater degree instead perhaps. Furthermore, most studies have additionally featured negligible effects from the loss of GM, as FC disturbances were still identified across the disorders regardless of structural GM or WM integrity thus implicating that FC changes likely underlie cognitive performance decline, with an Alzheimer’s disease/MCI study suggesting that the aberrations may even augment structural deficits.^205^ Therefore, there still arguably remain gaps in our knowledge concerning structural–functional relationships in terms of deciphering which occurs first within individuals to cause network disturbances or if indeed both occur simultaneously and thus could vary depending upon the disorder in question. The premise of these disturbances being found in individuals at high risk for AD and at prodromal MCI/svMCI disorder stages, further suggests that these changes in FC rs-fMRI outcome measures may provide potential biomarkers, which has previously been validated longitudinally with high reproducibility.^253^

Furthermore, whether the mPFC is vastly divergent from other prefrontal areas such as the dlPFC in terms of effects within dementia is potentially supported by a prevalence difference for each cognitive domain. Wong et al.^254^ interestingly reported that episodic memory deficits were found to underpin atrophy to marginally distinct prefrontal regions within behavioural variant FTD or Alzheimer’s disease subjects, respectively.^254^ Yet, it appears that further elucidation of the precise prefrontal contribution to differing cognitive domains and dementias has not been otherwise extensively explored.

Disruption in cholinergic signalling is a plausible mechanism for the altered PFC FC (discussed above). Learning and plasticity are supported and shaped by neurotransmitter systems in the brain. Cholinergic neurotransmission plays important roles in synaptic plasticity, glial remodelling and the regulation of inflammation. In addition, dopamine, and tonic GABAergic signalling^16^ amongst other neurotransmitters also play a critical role in learning and recovery following injury. Studies in animal models show that an intact cholinergic system ameliorates the effect of brain injury.^255^ The fornix is a key WM tract for memory function and fornix damage, in animal models and humans, impairs memory.^256^^,^^257^ However, combined fornix and cholinergic system lesions produce a quantitative deficit that exceeds the sum of the effects of the individual lesions.^256^ Fornix transection, performed *after* inferotemporal cholinergic depletion, produces the most severe deficit, markedly greater than the deficit when the order was reversed.^255^ The implication is that acetylcholine has an early but lasting effect on ameliorating the consequences of injury. The adaptive shift to alternative pathways to support memory function correlated with basal forebrain GM volume.^258^ Structural damage in the cholinergic system has been shown to be associated with a worse prognosis after traumatic brain injury.^259^ Neurotransmitter systems are likely to exert their effects on plasticity and learning through effects on synaptic plasticity, GM and WM structure, and FC. In the case of the cholinergic system, plasticity in the cortex is a likely mechanism. The reason for implicating the cortex is that the correlation was localized to the nucleus basalis of Meynert, which provides cholinergic innervation to the cortex but not the septum and diagonal band of Broca, which innervate the hippocampus. Such dysfunction in neurotransmitter signalling to both the cortex and hippocampus could affect PFC FC and underpin some of the cognitive deficits reported both for age and neurological conditions.

Nevertheless, it is still arguably difficult to ascertain confidently that the FC dysfunctions (Fig. 3) are valid, since practically all cross-sectional studies recruited small cohort sizes and took measurements over a relatively brief time frame, which possibly prevents causal relationships becoming inferable. Furthermore, extensive individual subject heterogeneity could limit valuable deductions between patient groups, as highlighted by Lee et al.^191^ showing diverse FC between good and poor cognitive performers in otherwise healthy aged individuals. The study subjects across the disorders may have also interestingly been affected by underlying vascular pathology, thereby potentially negating cross-study comparisons, as Vipin et al.^213^ suggested vascular factors cognitively impair PFC-associated networks such as the executive control network (glossary, Box 1) within MCI and Alzheimer’s disease subjects. Alternatively, the mPFC FC findings could be limited by this review design, since the deductions made are only qualitative and could have thus benefitted by implementing a more quantitative meta-analytical approach, so as to remove potential chance discoveries. To reflect the discrepancies of varied mPFC terminology in the field, several mPFC-specific search terms could have also been applied rather than repeatedly using ‘mPFC’, along with additional examples of large-scale networks so as to remove publication bias concerning the DMN. Experimental heterogeneity across studies due to variable patient recruitment, scan acquisition and data analytical techniques may have also resulted in several inconsistencies in detecting significant differences.^260^

## Conclusions and future directions

The role of the mPFC within cognition, ageing and dementia is diverse, yet seemingly fundamental for a range of critical cognitive operations. Animal work utilizing lesions and electrophysiology paired with behavioural tasks has refined our understanding by demonstrating precise mPFC subregions perform distinct executive functions. However, much of the equivalent rodent functions lack direct anatomical homology to primates, which implicates the lPFC instead. Such a disparity may reflect inconsistencies in mPFC terminology across studies as well as inadequacies controlling cognitively influential factors; therefore, further clarification of PFC terminology and rs-fMRI methodologies across the field is likely necessary.

Moreover, rodent models of ageing have exhibited substantial decline in the ability to perform tasks assessing mPFC-related executive functioning. Whilst rodent models of dementia-associated pathophysiological processes in PIS, VaD as well as additional neurodegenerative disorders have distinguished crucial inabilities to perform several behavioural paradigms tasking the mPFC. Studies examining large-scale brain connections have comprehensively shown that the mPFC’s links to heteromodal brain areas are integral for effectively coordinating numerous yet explicit cognitive paradigms, with the DMN exemplifying an extensively reported interconnectivity network that contains the mPFC as one of its central nodes. Existing rs-fMRI studies implicate mPFC FC variances during ageing and dementia-linked conditions between pairwise regions and globally within large-scale networks. Aberrations involving the DMN anterior and posterior sub-systems have been persistently reported across disorders, along with distinct patterns of neuropathological changes that may preclude structural defects and subsequent cognitive deterioration. In addition, some findings remain uncertain within the disorders due to methodological or sample inconsistencies, future validation could thus enable translation into effective biomarkers for earlier diagnosis or therapeutic intervention against pathological cognitive decline.

Accordingly, future work is essential for deciphering if the identified trends of reorganized FC intensity across disorders remain by replicating the rs-fMRI protocols whilst utilizing larger cohort sample sizes, which likely would remove any confounding effects of detected chance observations. Future rs-fMRI studies targeting the mPFC should also be conducted upon a longitudinal basis, which would enable a clearer understanding of how the disorders progressively worsen cognition through modifying mPFC FCs over the entire clinical course of an individual’s lifetime. This would clarify the remaining knowledge gap concerning structure-functional relationships and if FC changes definitively precede or even augment atrophy of the GM and WM tract changes, thereby further elucidating the underlying pathophysiological sequalae of these disorders. Moreover, conducting future rs-fMRI studies that focus specifically upon the impact of ageing and dementia-associated disorders within PFC-associated networks other than the DMN would better clarify how this important region is more broadly affected. Perhaps, future studies should also have a closer selection of individuals with comparable cognitive performances and clinical features that may further remove any patient heterogeneity effects responsible for divergent results. Detailed biochemical and molecular biology assessment of circuit-level receptors responsible mechanistically for the mPFC connectivity alterations would be most helpful, perhaps through use of agonists/antagonists targeting these receptors within rodent models, thereby further enabling the elucidation of refined pharmacological targets as a therapeutic intervention. Ultimately, use of network-based techniques may reconcile differences that remain within the field of mPFC cognitive function across species from a global integrated perspective by surveying the entire PFC and brain as a whole. Such deductions are particularly important considering the FCs between the mPFC with extra-frontal areas including the dlPFC are consistently implicated as necessary for cognition within both health and disease states.

## Data availability

Data sharing is not applicable to this article as no new data were created or analysed. The summarized data incorporated in the review are however available in Supplementary Table 1.

## Supplementary material

Supplementary material is available at *Brain Communications* online.

## Funding

This work was supported by previous grants from the Alzheimer’s Research UK (ARUK PG2013–22) and Medical Research Council, UK (MRC, G0500247).

## Competing interests

The authors report no competing interests.

## Supplementary Material

fcab125_Supplementary_Data

## Abbreviations

5-CSRTT =: 5-choice serial reaction time task
ACC =: anterior cingulate cortex
APDs =: atypical Parkinsonian disorders
BA =: Brodmann area
DMN =: default mode network
dmPFC =: dorsomedial prefrontal cortex
dlPFC =: dorsolateral prefrontal cortex
FC =: functional connectivity
FTD =: frontotemporal dementia
GM =: grey matter
ICA =: independent component analysis
ilPFC =: infralimbic prefrontal cortex
LPC =: lateral parietal cortex
lPFC =: lateral prefrontal cortex
MD =: medial dorsal nucleus of the thalamus
MO =: medial orbital cortices
mPFC =: medial prefrontal cortex
MTL =: medial temporal lobe
MCI =: mild cognitive impairment
MSA =: multiple system atrophy
OFC =: orbitofrontal cortex
PIS =: post-ischaemic stroke
PCC =: posterior cingulate cortex
PFC =: prefrontal cortex
plPFC =: prelimbic prefrontal cortex
PSP =: progressive supranuclear palsy
rs-fMRI =: resting-state functional magnetic resonance imaging
svMCI =: subcortical vascular mild cognitive impairment
VaD =: vascular dementia
vmPFC =: ventromedial prefrontal cortex
WM =: white matter
WCST =: Wisconsin Card Sorting Test
