# Supplementary material for: The role of the medial prefrontal cortex in cognition, ageing and dementia
Source: Brain Commun. 2021 Jun 11;3(3):fcab125. doi: 10.1093/braincomms/fcab125 (PMC8249104; doi:10.1093/braincomms/fcab125)
Supplement: fcab125_Supplementary_Data [file fcab125_Supplementary_Data.docx]

**Supplementary Material**

**Supplementary Table 1**: **Features of rs-fMRI in various studies showing the DMN**

| Disorder | Study | Total  Group  (N) | Disorder  Group (N) | Disorder  Female  (%) | Disorder  Age^a^  (Years) | Control  Group  (N) | Control  Female  (%) | Control  Age^a^  (Years) | Scanner | Method | Region/Network(s)  Investigated |
| --- | --- | --- | --- | --- | --- | --- | --- | --- | --- | --- | --- |
| Ageing | Lee *et al.* | 168 | 85 | 64.5 | 61.1 (3.9) | 83 | 56.6 | 34.6 (2.5) | 3 T S | VB | SN/DMN/CEN |
|  | Vidal-Piñeiro *et al.* | 50 | 25 | 68.0 | 68.9 (3.8) | 25 | 68.0 | 23.1 (2.0) | 3 T S | ICA | mPFC/PCU |
|  | Campbell *et al.* | 84 | 39 | 61.5 | 69.0 (5.2) | 45 | 48.9 | 22.4 (3.1) | 3 T S | SB | PHG/dmPFC/vPCC/  dPCC |
|  | Andrews-Hanna *et al.* | 93 | 55 | 67.3 | 76.5 (8.2) | 38 | 52.7 | 22.4 (3.6) | 1.5 T S | SB | mPFC/PCU/LPC/  HF |
|  | Onoda *et al.* | 73 | N/A | 41.1 | 60.2(12.8) | N/A | 41.1 | 60.2 (12.8) | 1.5 T S | ICA/SB | DMN/FPN/VN/TN/CN |
|  | Wu *et al.* | 40 | 22 | 54.5 | 69.8 (5.8) | 18 | 50.0 | 23.9 (1.8) | 1.5 T GE | SB | PCC/vmPFC |
|  | Shu *et al.* | 29 | 7 | 57.1 | 70.1 (4.9) | 22 | 54.5 | 71.8 (3.8) | 1.5 T GE | SB | HFN |
|  | Sheline *et al.* | 100 | 38 | 23.7 | 58.8 (8.5) | 62 | 30.6 | 61.6 (8.1) | 3 T S | SB | PCU |
|  | Hedden *et al.* | 38 | 21 | N/A | 73.4 (7.9) | 17 | N/A | 72.8 (8.1) | 3 T S | SB | PCC |
|  | Sheline *et al.* | 68 | 20 | 60.0 | 72.7 (6.5) | 48 | 66.7 | 73.6 (6.6) | 3 T | SB | PCU |

***Supplementary Table 1 (Continued)***

| Disorder | Study | Total  Group  (N) | Disorder  Group (N) | Disorder  Female  (%) | Disorder  Age^a^  (Years) | Control  Group  (N) | Control  Female  (%) | Control  Age^a^  (Years) | Scanner | Method | Region/Network(s)  Investigated |
| --- | --- | --- | --- | --- | --- | --- | --- | --- | --- | --- | --- |
| MCI  or AD | Yue *et al.* | 48 | 26 | 38.5 | 72.4 (5.1) | 22 | 45.5 | 72.8 (3.5) | 1.5 T GM | SB | pPHG |
|  | Qi *et al.* | 28 | 14 | 57.1 | 71.8 (7.3) | 14 | 42.9 | 70.4 (5.8) | 3 T S | ICA | DMN |
|  | Cai *et al.* | 77 | 39 | 51.3 | 72.4 (5.0) | 38 | 50.0 | 73.9 (3.9) | 3 T P | VB | DMN/LS/SMN/VN |
|  | Scherr *et al.** | 53 | 35 | 51.4 | 66.3 (8.1) | 18 | 44.4 | 63.2 (8.5) | 2 T* S | ICA | DMN |
|  | Jin *et al.* | 16 | 8 | 37.5 | 60.9 (3.2) | 8 | 50.0 | 60.6 (8.3) | 3 T GE | ICA | DMN |
|  | Bai *et al.* | 56 | 30 | 50.0 | 72.5 (4.4) | 26 | 53.8 | 71.6 (5.3) | 1.5 T GE | SB | PCC |
|  | Gardini *et al.* | 42 | 21 | 38.1 | 70.6 (4.7) | 21 | 66.7 | 69.8 (6.5) | 3 T GE | SB | mPFC/PCC |
|  | Sorg *et al.* | 40 | 24 | 45.8 | 69.3 (8.1) | 16 | 37.5 | 68.1 (3.8) | 1.5 T S | ICA/SB | DMN/PCC/MTL |
|  | Gili *et al.** | 31 | 21 | 38.1 | 71.6 (6.0) | 10 | 30.0 | 64.1 (10.5) | 3 T S | ICA/SB | DMN/PCC/mPFC |
|  | Vipin *et al.** | 248 | 183 | 54.6 | 74.4 (7.6) | 65 | 55.4 | 67.3 (6.2) | 3 T S | SB | DMN/ECN |
|  | Dai *et al.*^b^ | 70 | 32 | 56.3 | 71.3 (8.6) | 38 | 65.8 | 68.4 (7.8) | 3 T S | VB/GT | WB |
|  | Agosta *et al.** | 38 | 25 | 44.0 | 71.8 (8.6) | 13 | 61.5 | 68.5 (6.9) | 1.5 T S | ICA | DMN/FPN/ECN/SN |

***Supplementary Table 1 (Continued)***

| Disorder | Study | Total  Group  (N) | Disorder  Group (N) | Disorder  Female  (%) | Disorder  Age^a^  (Years) | Control  Group  (N) | Control  Female  (%) | Control  Age^a^  (Years) | Scanner | Method | Region/Network(s)  Investigated |
| --- | --- | --- | --- | --- | --- | --- | --- | --- | --- | --- | --- |
| svMCI  or PIS | Sun *et al.* | 34 | 16 | 12.5 | 69.1 (7.8) | 18 | 11.1 | 66.2 (7.7) | 3 T P | SB | PCC |
|  | Yi *et al.* | 47 | 21 | 57.1 | 65.9 (9.8) | 26 | 53.8 | 64.8 (8.1) | 3 T S | GT | DMN/VN/AN/  SMN |
|  | Zhou *et al.* | 55 | 32 | 56.2 | 70.1 (8.3) | 23 | 39.1 | 68.9 (7.1) | 3 T GE | SB | mPFC/THA |
|  | Yi *et al.* | 54 | 26 | 57.7 | 66.7 (9.5) | 28 | 57.1 | 65.3 (8.1) | 3 T S | VB | mPFC/PCC/PCU/  IPL |
|  | Jiang *et al.*^b^ | 46 | 21 | 28.6 | 65.1(11.3) | 25 | 28.0 | 66.7 (7.2) | 3 T P | ICA | DMN |
|  | Zhu *et al.*^b^ | 57 | 29 | 48.3 | 57.6 (0.2) | 28 | 50.0 | 56.9 (0.5) | 3 T GE | GT | PCU/MFG/ANG/  SFGmed |
|  | Dacosta-Aguayo *et al.*^b^ | 28 | 11 | 27.3 | 61.9 (7.9) | 17 | 35.3 | 63.8 (3.6) | 3 T S | ICA/SB/GT | DMN |
|  | Ding *et al.*^b^ | 38 | 18 | 44.4 | 63.9 (9.2) | 20 | 45.0 | 62.9 (9.3) | 3 T P | ICA | DMN |
|  | Park *et al.*^b^ | 22 | 11 | 27.3 | 55.7 (8.7) | 11 | 18.2 | 56.2 (2.9) | 3 T P | ICA/SB | DMN/PCC/mPFC/  IPL |
|  | Tuladhar *et* al.^b^ | 41 | 20 | 35.0 | 55.1(11.8) | 21 | 47.6 | 51.3 (13.7) | 1.5 T S | ICA/VB | DMN |

***Supplementary Table 1 (Continued)***

| Disorder | Study | Total  Group  (N) | Disorder  Group (N) | Disorder  Female  (%) | Disorder  Age^a^  (Years) | Control  Group  (N) | Control  Female  (%) | Control  Age^a^  (Years) | Scanner | Method | Region/Network(s)  Investigated |
| --- | --- | --- | --- | --- | --- | --- | --- | --- | --- | --- | --- |
| PD  or APDs | Disbrow *et al.* | 34 | 14 | 50.0 | 65.9 (5.3) | 20 | 40.0 | 66.7 (4.7) | 3 T S | SB | ECN/DMN |
|  | Seibert *et al.* | 35 | 18 | 11.1 | 72.0 (7.0) | 19 | 57.9 | 76.0 (9.0) | 1.5 T GE | SB | IC/Caudate |
|  | Ghahremani *et al.* | 120 | 60 | 66.7 | 62.3 (N/A) | 60 | 77.7 | 63.2 (N/A) | 3 T | SB | PCC/mPFC/TPL/  LPC |
|  | Thibes *et al.* | 92 | 55 | 23.6 | 64.5 (12.5) | 37 | 73.0 | 63.8 (11.1) | 3 T S | SB | mPFC/PCU |
|  | Lucas-Jiménez *et al.* | 53 | 37 | 40.5 | 68.0 (6.2) | 16 | 25.0 | 65.1 (6.8) | 3 T P | SB | PCC/ACC/mPFC/  MTL |
|  | Kawabata *et al.*^b^ | 64 | 32 | 43.8 | 63.4 (7.8) | 32 | 43.8 | 63.4 (7.6) | 3 T S | ICA/SB | ECN/SN/CER |
|  | Rosskopf *et al.^b^* | 69 | 34 | 47.1 | 69.9 (7.7) | 35 | 48.6 | 66.7 (6.3) | 1.5 T S | SB | DMN/MB/MC |
| FTD | Caminiti *et al.* | 42 | 12 | 25.0 | 63.1  (7.2) | 30 | 40.0 | 58.8  (7.3) | 3 T P | ICA | DMN/ATN/  dmPFC |
|  | Bejanin *et al*. | 52 | 13 | 57.9 | 66.6  (7.0) | 39 | 51.3 | 68.9  (7.0) | 3 T P | SB/VB | AMG/mPFC/ACC/PCC |

The studies listed here involve subjects over the age of 50 years old and do not include use of independent component analysis (ICA) or voxel-wise, seed-based and graph theory rs-fMRI methods that did not assess mPFC FC between aged participants or with a disorder and age-matched cognitively unimpaired and/or healthy controls. They were also not included if there were no significant differences between groups in mean connectivity to other brain regions with the difference trends. The region/network(s) investigated column shows the location of the seed for each of the seed-based studies. ^a^Age is displayed as mean (standard deviation), ^b^Studies investigating AD or PIS or APDs respectively and *Studies investigating both AD dementia and MCI cohorts. *Abbreviations: AD, Alzheimer’s disease; AMG, amygdala; ANG, angular gyrus; ACC, anterior cingulate cortex; ATN, attentional network; APDs, atypical Parkinsonian disorders; AN, auditory network; CEN, central executive network; CER, cerebellum; CN, cerebellum network; DMN, default mode network; d, dorsal; dmPFC, dorsomedial prefrontal cortex; ECN, executive control network; FPN, fronto-parietal network; FTD, frontotemporal dementia; GE, General Electrics; GT, graph theory; HF, hippocampal formation; HFN, hippocampal formation network; ICA, independent component analysis; IPL, inferior parietal lobule; IC, isthmus cingulate; LPC, lateral parietal cortex; LS, limbic system; mPFC, medial prefrontal cortex; MTL, medial temporal lobe; MB, midbrain; MFG, middle frontal gyrus; MC, motor cortex; MCI, mild cognitive impairment; PHG, parahippocampal gyrus; PD, Parkinson’s disease; P, Philips; p, posterior; PCC, posterior cingulate cortex; PIS, post-ischaemic stroke; PCU, precuneus; SN, salience network; SB, seed-based; SMN, sensorimotor network; S, Siemens; svMCI; subcortical vascular mild cognitive impairment; SFGmed, superior frontal gyrus medial; TN, temporal network; TPL, temporal parietal lobe; T, Tesla; THA, thalamus; v, ventral; vmPFC, ventromedial prefrontal cortex; VN, visual network; VB, voxel-based; WB, whole brain.*
